# Epistemology for Beginners: Two- to Five-Year-Old Children's Representation of Falsity

**DOI:** 10.1371/journal.pone.0140658

**Published:** 2015-10-20

**Authors:** Olivier Mascaro, Olivier Morin

**Affiliations:** 1 Jean Nicod Institute, Paris, France; 2 Cognitive Development Center, Central European University, Budapest, Hungary; 3 Laboratoire sur le Language, le Cerveau et la Cognition, L2C2, CNRS/Lyon1 University, UMR5304, Lyon, France; 4 Social Mind Center, Central European University, Budapest, Hungary; University of Akron, UNITED STATES

## Abstract

This paper investigates the ontogeny of human’s naive concept of truth. Surprisingly, children find it hard to treat assertions as false before their fifth birthday. Yet, we show in six studies (N = 140) that human’s concept of falsity develops early. Two-year-olds use truth-functional negation to exclude one term in an alternative (Study 1). Three-year-olds can evaluate discrepancies between the content of a representation and what it aims at representing (Study 2). They use this knowledge to treat beliefs and assertions as false (Study 3). Four-year-olds recognise the involutive nature of falsity ascriptions: they properly infer ‘p’ from ‘It is not true that “It is not true that “p””‘ (Study 4), an inference that rests on second-order representations of representations. Controls confirm that children do not merely equate being mistaken with failing to achieve one’s goal (Studies 5 and 6). These results demonstrate remarkable capacities to evaluate representations, and indicate that in the absence of formal training, young children develop the building blocks of a theory of truth and falsity—a naive epistemology. We suggest that children’s difficulties in discarding false assertions need not reflect any conceptual lacuna, and may originate from their being trustful.

## Introduction

Truth has baffled philosophers for centuries. We are still lacking a theory that would unify the range of epistemic intuitions elicited by falsity and truth, by facts and by the way propositions stick to them (or fail to do so). This in itself would be a reason to doubt that proficiency in dealing with epistemological issues comes early in human ontogeny. Moreover, whether children under age four can even represent the kind of representations that can be false—like beliefs—is debated [1–9]. None of this suggests that young minds should be capable of handling truth or falsity concepts. Yet this paper shows that before receiving any formal training young children possess an incipient concept of falsity.

As this is not a work of normative epistemology, we did not feel the need to stick to one of the canonical philosophical theories of truth. We shall merely make two assumptions. First, to believe that a proposition like 'the cat is on the mat' is false requires to represent that proposition without accepting it [10], i.e., to form a secondary representation [11]. Second, to believe that 'the cat is on the mat’ is false, is to evaluate the relationship between a representation (the proposition) and what it represents (e.g. a state of the world). One thus forms a representation of a relation of representation, in other words a ‘metarepresentation’ [11–13].

### An empirical paradox: Children’s Competence and Difficulties in Treating Assertions as False

Before age five, children’s assessment of falsity is characterised by a blend of surprising competence and remarkable difficulties. This paradoxical pattern is most evident in the way three-year-old children process false assertions.

Three-year-olds mistakenly interpret outdated signals as if they were still accurate [14–16]. When asked merely to memorise and repeat false assertions, they tend to correct their report of what speakers said as if that message had been true [17]. These difficulties cannot be reduced to a lack of mastery of ‘that-clauses’ (e.g., ‘John says *that* the earth is flat’), since they do not extend to cases in which three-year-olds are asked to report what someone wants [18]. Moreover, three-year-olds have surprising difficulties in rejecting false assertions. They fail to mistrust a malevolent communicator who has been misleading them (with pointing or verbal testimony), for up to 10 repeated trials [19–23]. This in spite their well-established capacity to trust nice over mean informants [22, 24–27]. Children under four years old also fail to reject what is communicated by a single informant who has a false belief [28], despite their capacity to interpret what a speaker says on the basis of her false beliefs [29–30].

Around the ages of four to five, children become proficient at interpreting, memorising and rejecting false assertions. These changes co-occur with an increase in children’s capacity to predict explicitly a character’s behaviour on the basis of her false belief [15, 17, 23, 28, 31]. From this evidence, it would be tempting to conclude that the ability to represent veracity emerges during children’s fifth year of life, along with a full-blown capacity to represent beliefs. Yet, data suggest the contrary. By toddlerhood, children’s remarkable difficulties in assessing the truth and falsity of some representations co-exist with unexpected areas of proficiency.

Consider the following example:

‘*Child 21 months*

C sitting at table with cup of milk. Looks at observer, points to cup.

*C to observer*: Beer! (laughs). Beer no!’

([32], p.161).

In this case, a 21-month-old child appears to play—effortlessly—with the discrepancy between words and reality. This type of joke, typically emerging around age two [33], is not the only sign of incipient sensitivity to the accuracy of assertions. Event-related brain potentials [34–36] and looking time studies [37–38] show that infants detect the use of inaccurate labels for familiar objects. From their second year of life on, children also contradict adults who mislabel things, often using negation in correcting them [37, 39–43]. By age three, these corrections are sensitive to the type of speech act performed by speakers: three-year-olds are more likely to direct their protest at speakers who assert something false, than at those who give unobeyed orders [44]. Three-year-olds can also explicitly tell that speakers who were factually incorrect were ‘not very good at answering questions’ or were ‘wrong’ [45–49]. This sensitivity to inaccuracy has well-documented effects on children’s trust. Toddlers and three-year-olds lower their trust in previously accurate informants, [45–46, 50–57]; and they prefer to learn what an informant says if a third party assents to it, rather than dissents [58–59].

In short, two- to three-year-olds are sensitive to the accuracy of assertions, to a point. Their difficulties in interpreting false signs, memorising false utterances, or in rejecting misleading assertions are all the more remarkable. Below, we outline three hypotheses that could explain this paradoxical blend of competence and difficulties.

### How Do Children Represent Inaccuracy?

Truth and falsity assessments have two central characteristics [60]. First, assessments of truth have an intension, which characterises *what it means* for a proposition to be true or false. Second, truth and falsity assessments have an extension, which specifies *what* can be true or false. Young preschoolers’ paradoxical grasp of truth and falsity could be explained by difficulties at any of these two levels.

#### The Wrong Intension Hypothesis

A first possibility is that children before age four fail to grasp what it means for a proposition to be false. Instead, they would assess communicative actions and informants using other cognitive capacities. Falsity is just one of the many ways an utterance can be inadequate. An utterance could also be uninformative, too difficult to process, or linguistically improper. Here, we focus on informativeness (but see S1 Text for a more detailed argument addressing other criteria that can be used to evaluate utterances).

Vervet and rhesus monkeys respond to their conspecifics' calls, but they are less responsive when the caller repeatedly produced misleading calls in the past [61–62]. Should we assume that these monkeys’ treat such alarm calls as false? Not necessarily. A cognitive system can block learning from certain sources without attaching a ‘true’ or a ‘false’ tag to the representations that it handles. The trick is simply to learn to use certain sources of information but not others [63–64]. Likewise, children could treat inaccurate assertions as uninformative rather than false. This would give them reason enough to correct inaccurate informants and rely on them less. This hypothesis would explain why children find it hard to interpret [15] and to memorise [17] inaccurate utterances. It could also explain why children trust misleading informants when no alternative source of information is present [19–23]: a piece of evidence, even of low value, is better than nothing.

The Wrong Intension hypothesis can be empirically distinguished from a genuine evaluation of falsity. By recognising that ‘p’ is false, I can infer not-p [65]. Conversely, treating ‘p’ as uninformative, hard to process, or linguistically inappropriate, tells me nothing about not-p. Thus, if the Wrong Intension hypothesis is correct, children should ignore the content of false representations. By contrast, if children can represent falsity, they should recognise that if “p” is false, then not-p is the case.

#### The Wrong Extension Hypothesis

A second possibility is that children have some incipient notion of falsity, but fail to recognise that certain representations, such as beliefs, or assertions, can be false (the Wrong Extension Hypothesis). Children would recognise that the content of representations such as utterances can be at odds with reality well before the age of four; this would allow them to understand pretence, among other things. Young children’s difficulty would lie in recognizing that what is not the case can be taken to be the case, a capacity sometimes thought to be required for representing false beliefs and false assertions [11,66]. This view posits that before four, children would fail to recognise that one can believe or assert something false, therefore explaining three-year-olds’ difficulties in interpreting false assertions, memorising them, or treating them with skepticism.

#### The Optimist Epistemologist Hypothesis

A third possibility is that young children can treat assertions and beliefs as false, but that this ability is masked by other dispositions. We suggest that young children sometimes fail to treat representations as false because they are trusting—the Optimist Epistemologist Hypothesis.

The view that assertions carry a specific kind of epistemic warrant is a mainstay of the epistemology of testimony [67]. According to a popular thesis, the fact that a proposition has been asserted is in itself a reason to believe it. Whether that thesis is normatively true is not a question we need to address here; but it may have a counterpart in laypeople's intuitions, especially in young children [20–21, 68–72].

It has often been noted that children depend on communication to an inordinate extent [73–74]. Two factors should foster an optimistic outlook on social epistemology in young children. First, younger children have accumulated less evidence and knowledge than older children or adults have. As a consequence, they have less reason to discard testimonies that contradict what they believe. Second, young children have little choice but to trust a reduced number of caregivers to provide them with what they need, be it food, physical comfort, or information. This dependency makes mistrust a costly option [22, 75].

If children are Optimist Epistemologists, their difficulties processing false assertions need not spring from any conceptual lacuna. An expectation that communication is generally reliable could make children more likely to reinterpret misleading signals as accurate [15] and to distort their memory of false utterances in a way that makes them true [17]. This hypothesis would also explain why children are often blind to the possibility of being misinformed by a liar or by a mistaken informant [20–23].

To judge between our three hypotheses, we tested two- to five-year-old children’s representations of falsity. To anticipate, data were inconsistent with the wrong intention and the wrong extension hypotheses, and suggest that humans’ intuitive representations of truth may emerge early during ontogeny. Two-year-olds use truth-functional negation to exclude one term in an alternative (Study 1). Three-year-olds can evaluate the discrepancy between the content of a representation and what it aims at representing (Study 2), and they apply this knowledge to the content of beliefs and assertions (Studies 3 and 5). Four-year-olds recognise the involutive nature of falsity ascription: they properly infer ‘p’ from ‘It is not true that “It is not true that “p””‘ (Studies 4 and 6). Overall, data are consistent with the optimist epistemologist hypothesis: young children seem able to treat assertions and beliefs as false. Trust in informants may explain why they often fail to make use of this ability.

## General Methods

### Ethics statement

This research was approved by the institutional review board of the doctoral school ED3C (Ecole Normale Supérieure, Ecole des Hautes Etudes en Sciences Sociales, University Paris VI) and by the regional board of schools (Inspection Académique du Tarn). It was conducted in accordance with the ethical guidelines of the French National Research Center (CNRS). The participants’ parents provided written informed consent.

### Participants

Children were recruited in schools of mid-sized French cities (10000 to 60000 inhabitants). In each school, all children whose parents gave informed consent were tested, thus explaining sample size variations across studies.

### Materials and Setting

The tests took place in the children’s school or day-care centre, in a quiet room. The experimenter and the participating child sat facing each other across a small table. A coin, pairs of boxes of various colours, sizes and shapes, and different animal puppets were used in the hiding games reported in Studies 1 to 6. Coloured pencils and a box of smarties were used in the unexpected content false belief task [76]. Two human puppets, a marble, and a small box and basket were used in the unexpected transfer false belief task [77].

### Hiding Tasks’ Procedure

The same general procedure was used in all the hiding tasks (exclusion task, false assertion task, true assertion task, false belief task, true belief task, first-order falsity task and second-order falsity task). Two opaque boxes of different colours were placed on the experimental table, each equally distant from the child, one on the left side of the table, and the other on the right side of the table. Before the hiding game started, the experimenter told the child: ‘*Let’s play a game*! *I am going to hide this coin in one of these boxes*, *and you have to find it*, *okay*?’, while showing the coin, and pointing successively towards each of the test boxes. Then the experimenter asked the child: ‘*Turn around while I hide the coin*’. Once the child had turned, the experimenter hid the coin in a bag below the experimental table. The boxes remained closed and empty. After hiding the coin, the experimenter said: ‘*That’s OK*, *I hid the coin*!’ and the game started. The remaining part of the hiding tasks is detailed below for each particular study. The following were randomised across trials: The side of the box corresponding to the correct answer (right or left of the table); for each pair of boxes, which one of the two boxes corresponded to the correct answer. At the end of a trial, if an additional trial of the same task was to take place, the experimenter said: ‘*Let’s play again*!’ while replacing the boxes used in the previous trial with two new boxes. A different pair of boxes was used for each trial. The children did not receive feedback on their answers to any of the test questions, and were never told where the coin was hidden. Post-hoc analyses on the effect of order of presentation on test questions confirmed that this manipulation was effective: there was no order effect on performance in Studies that used repeated measures (all *p*s >.21).

### Data Coding and Analysis

The children answered verbally or by pointing. Data were coded online by the experimenter (as in previous studies using similar tasks, e.g. [19–23]). All the statistical tests reported in this paper are two-tailed.

## Study 1

### Methods

#### Participants

Thirteen two-year-olds (*n* = 13; *M* = 2;7, range 2;4 to 2;10) participated. Three additional children were excluded because they did not understand the purpose of the game in the familiarisation phase.

#### Procedure

Children were tested in the presence of a familiar caregiver. They participated in a familiarisation, and later were introduced to hiding games in which they had to find a coin that could be hidden in one of two boxes, as detailed in the General Methods section.

#### Familiarisation

A familiarisation, adapted from Behne, Carpenter, and Tomasello [78] ensured that children understood the hiding games. Children were facing the experimenter across a small table on which two boxes were placed. The experimenter opened the boxes, and said: ‘*Look*, *I’ll hide the coin*’. He placed the coin in one of the boxes in full view of the participant, closed the boxes, and then asked: ‘*So*, *where is the coin*?’. This familiarisation procedure was repeated four times before the test began. To be included in the final data analysis, children had to find the coin in at least the last two warm-up trials.

#### Exclusion Task

This task was adapted from the ‘Disjunction test’ of Mascaro and Sperber [22]. It involved no puppet. The experimenter acted as if hiding a coin in one of two boxes as indicated in the General Methods section. Then, the experimenter himself informed the child. He pointed to a box (e.g. the red box), while saying: ‘*The coin is not in the {colour of the box*, *e*.*g*. *red} box*’. The child was then asked: ‘*So*, *where is the coin*?’. Children’s answers were considered correct if they pointed at or verbally indicated the box that was not designated by the experimenter.

#### False Assertion Task

In this task an animal puppet was used as an informant (e.g., a bear). After pretending to hide the coin in one of the boxes as indicated in the General Methods section, the experimenter said: ‘*Now the {name of the puppet*, *e*.*g*. *bear} will say something to you*’. The puppet, manipulated by the experimenter, approached one of the boxes, and patted its lid, while the experimenter said in a distinctive voice: ‘*The coin is in the {colour of the box*, *e*.*g*. *white} box*’. Then, the experimenter replaced the puppet in the centre of the table, at equidistance from the boxes and said: ‘*The {name of the puppet*, *e*.*g*. *bear} says that the coin is in the {colour of the box*, *e*.*g*. *white} box*, *but it’s not true*! *It’s not true that the coin is in the {colour of the box*, *e*.*g*. *white} box*!’. Following this commentary, the experimenter asked the test question: *‘So*, *where is the coin*?*’*. Children’s answers were considered correct if they selected the box that was not indicated by the puppet.

#### Design

Each participant was presented with an exclusion task, and with a false assertion task (order of presentation counterbalanced: 6 children were presented first with the falsity task, and 7 children were presented first with the exclusion task). Different pairs of coloured boxes were used in the warm-up phase, in the false assertion task, and in the exclusion task.

### Results

The participants’ performance on the exclusion task was significantly higher than their performance on the false assertion task (eight children succeeded on the exclusion task while failing on the false assertion task, no child did the opposite; *p* = .013, McNemar test). Two-year-olds had a tendency to follow the advice of the puppet in the false assertion task—(10 children out of 13 did so, *p* = .09, two-choice binomial test). Conversely, 11 of 13 participants correctly selected the box that was not pointed at in the exclusion task (*p* = .02, two-choice binomial test).

### Discussion

In the exclusion task, two-year-olds used negation to exclude one location, and select the other. Merely ignoring what was negated was not sufficient to succeed in the task. This first result dovetails with studies suggesting that children understand truth-functional negation around their second birthday [79]. An anonymous reviewer suggested that children may succeed in our task by avoiding the box about which a negated statement was made, perhaps assuming that the experimenter ordered them to avoid one box. Although nothing in our design was set to elicit an imperative interpretation, we cannot rule out this alternative hypothesis entirely. Yet, previous studies suggest that to elicit avoidance of a location in two-year-olds using imperative statements is not easy. For example, two-year-olds are at chance in avoiding a box that an experimenter explicitly forbid them to select (by saying to the child: “No, don’t take this one”) [80]). By contrast, in our test children consistently selected the correct box when told where the toy was not. In our view, this success is best explained by assuming that children interpreted the experimenter’s comment as a declarative sentence, indicating where the coin was not (in line with their capacity to produce and interpret negation in other contexts, e.g. [40,81–82]).

Prima facie, our exclusion task may seem to suffice for showing that children, on top of excluding one box, perform a disjunctive inference (i.e., they infer that if a reward can be in A or B, and is not in A, then it is in B). However that is not the case. Let’s imagine that after hiding, the child assumes that the reward has equivalent chances to be in container A or in container B (*p*(A) = *p*(B) = 0.5). Evidence for a disjunctive inference can be established if, upon discovering that the coin is not in A, children increase their estimate of the probability of the reward to be in B (e.g., assume that *p*(B) = 1). Our task does not provide evidence for this inferential process, because children could still succeed without it. For example, if children think that the reward has a probability of 0 to be in A, and a probability of .5 to be in B, that is sufficient for them to select B over A (see [83–85], for proposals addressing this issue).

Despite their success in the exclusion task, two-year-olds tend to trust a testimony that is said to be ‘not true’. These difficulties could stem from a lack of understanding of the word ‘true’ before age three [47]. In Study 2, we tested the developmental onset of children’s capacity to interpret explicit comments about the truth or falsity of a proposition.

## Study 2

### Methods

#### Participants

Thirty-two three-year-olds participated (*M* = 3;3, range 2;10 to 3;11). Two additional children failed to understand the purpose of the game and were excluded from analysis.

#### False Assertion Task

This task was similar to the false assertion task of Study 1 except that no familiar caregiver accompanied the child in the testing room. Moreover, unlike in Study 1, no familiarisation preceded the false assertion task of Study 2.

#### True Assertion Task

The true assertion task was similar to the false assertion task, except for the experimenter’s commentary on what the puppet said. After the coin was said to be hidden, and the puppet testified (saying e.g. ‘*The coin is in the white box*’), the experimenter confirmed the puppet’s testimony, by saying: ‘*The {name of the puppet*, *e*.*g*. *giraffe} says that the coin is in the {colour of the box*, *e*.*g*. *white} box*, *and it’s true*! *It’s true that the coin is in the {colour of the box*, *e*.*g*. *white} box*!’. Children’s answers in the true assertion task were considered correct if they selected the box indicated by the puppet.

#### Standard False Belief Task

Children were presented with an unexpected content task ("smarties" task), using the procedure of Leslie, Perner, Frith & Leekam [76]. We included this task to compare the development of children’s capacity to represent representations in a standard false belief task (the unexpected content task), and in our false assertion task. The unexpected content tasks also allowed us to check that our sample of children was not exceptionally precocious in forming explicit representations of representations.

#### Design

All children were presented with a false assertion task followed by a false belief task. A subset of participants (*n* = 17, *M* = 3;6, range 2;10 to 3;9) was also tested on a true assertion task at the end of the session. For children who were tested both on the false assertion task and true assertion tasks, different puppets were employed in each task.

### Results

Almost all children followed the advice of the puppet in the true assertion task (16 out of 17 participants, *p* = .0003, two-choice binomial test). Conversely, the majority of children selected the box not indicated by the puppet in the false assertion task (23 out of 32 participants, *p* = .02, two-choice binomial test). Children’s tendency to follow the advice of the puppet was higher in the true assertion task than in the false assertion task (11 children followed the advice of the puppet in the true assertion task, and did not do so in the false assertion task, while no child showed the opposite pattern of answers, *p* = .003, McNemar test). Only 16% (5/32) of participants passed the standard false belief task. Children were more likely to suceed on the false assertion task than on standard false belief tasks (19 children succeeded on the false assertion task while failing on the standard false belief task, 1 child did the opposite, *p* = .0001, Mc Nemar test). Notice that since our primary interest was in children’s performance in the false assertion tasks, they were always presented before the standard false belief task. We controlled for potential worries related to this fixed order in our subsequent study (Study 3), by counterbalancing the presentation of false assertion tasks and of standard false belief tasks.

### Discussion

In Study 2, children as young as three were able to treat propositions as false. Doing so requires representing a representation whose content one does not accept. Moreover, our results confirm that three-year-old children can assess the mismatch between a representation and what it aims at representing—in our task, reality,—a signature of the capacity to metarepresent [10,11]. Thus, Study 2 refutes the Wrong Intension hypothesis. Had children assumed that what is ‘not true’ is not worth processing, they would have ignored the testimony of the puppet, and should have selected one of the boxes at chance in the falsity task. Instead children expected the content of the puppet’s false testimony to depart systematically from reality.

When no alternative source of information is present, children under five have remarkable difficulties to infer that a misleading or mistaken informant will tell them something false [19–23]. As our results indicate, these difficulties do not stem from inabilities to represent falsity. Rather, what may emerge around five is a heightened sensitivity to the possibility of being misinformed. How did children manage to overcome their trust in our study? Quite simply because they did not face a single informant, but two. In our task, the experimenter straightforwardly contradicted the puppet’s testimony by saying that it was ‘not true’. Children were thus placed in a situation in which they had to choose between trusting the puppet, and the experimenter. Given that the puppet was manipulated by the experimenter himself, children had every reason to trust the experimenter. Crucially, to interpret the experimenter’s comment, children had to treat the content of the puppet’s testimony as false.

In our false assertion task, the experimenter repeated the content of the puppet’s assertion, and embedded it in a single proposition (e.g. by saying ‘it is not true that the coin is in the red box’). Therefore, children could have succeeded in Studies 1 and 2 by assessing the veracity of propositions, without necessarily recognizing that these propositions were asserted. Study 3 controlled for this possibility by allowing children to learn what a character asserted or believed, and telling them that the character was mistaken.

## Study 3

### Methods

#### Participants

18 three-year-old children participated in the study (*M* = 3;7, range 3;1 to 4;0). Two additional participants refused to participate to the full experimental session and were excluded from analyses.

#### False Assertion Task

This task was similar to Study 2’s false assertion task. However, the experimenter commented on the veracity of the puppet’s testimony by saying: *‘The {name of the animal puppet*, *e*.*g*. *frog} says that the coin is in {colour of the box indicated by the puppet*, *e*.*g*. *white} box*, *but he/she* ^*_*^ *is mistaken*!’ (the French verb that we used was ‘*se tromper*’, literally "to be mistaken"). This comment was followed by the test question (*‘So*, *where is the coin*?*’)*. Correct answers consisted in selecting the box that was not indicated by the puppet. A “memory of representation” question (‘*Where did the frog say that the coin was*?’) and a “memory of behaviour” question (‘*Which box did the frog touch*?’) followed the test question (order of presentation counterbalanced across trials). Correct answers on the memory questions consisted in selecting the box that had been touched by the puppet. Children were presented with two trials of the false assertion task without feedback, using the same puppet as an informant. For each question of the false assertion task, children’s scores on these two trials were added to yield a score ranging from 0 to 2.

#### False Belief Task

This task was similar to the false assertion task of Study 3, except that the puppet did not communicate. After the coin was hidden, the experimenter explained: ‘*Now*, *the puppet will try to find the coin*’. He manipulated the puppet as if it were reaching for one of the boxes. Then, the experimenter replaced the puppet in the centre of the table, equidistant from the boxes. He explained: ‘*The {name of the animal puppet*, *e*.*g*. *giraffe} believes that the coin is in the {colour of the box indicated by the puppet*, *e*.*g*. *white} box*, *but he/she is mistaken*!’. The test question was then asked: ‘*So*, *where is the coin*?’. Correct answers consisted in selecting the box that had not been touched by the puppet. The experimenter also asked a ‘memory of representation’ question (‘*Where did the giraffe believe that the coin was*?’) and a ‘memory of behaviour’ question (*‘Which box did the giraffe touch*?’) (order of presentation counterbalanced across trials). Correct answers on memory questions consisted in selecting the box that was touched by the puppet. The false belief task was presented for two trials without feedback, using the same puppet in the two trials. For each question of the false belief task, the children’s scores on these two trials were added to yield a score ranging from 0 to 2.

Typically, standard false belief tasks require the child to infer the content of a belief, based on observable cues (what a character did or did not perceive, for instance). By contrast, in our task children are directly told about the content of a character’s belief (without knowing what the origin of this belief is). Yet, we named our task ‘false belief’ task following the convention established by other researchers [86]. This terminological choice seemed appropriate, given that in our task the experimenter told children what a character believed, and told them that it was mistaken.

#### True Assertion Task

This task was similar to the false assertion task of Study 3, except that the experimenter said that the puppet’s testimony was true: e.g. ‘*The {name of the animal puppet*, *e*.*g*. *bear} says that the coin is in the {colour of the box indicated by the puppet*, *e*.*g*. *white} box*, *and he/she is right*!’. Children’s answers to the test question (‘*So*, *where is the coin*?’) were considered correct if they selected the box that was indicated by the puppet.

#### True Belief Task

This task was similar to the false belief task of Study 3, except that this time the experimenter said that the puppet’s belief was true: e.g. ‘*The {name of the animal puppet*, *e*.*g*. *elephant} believes that the coin is in the {colour of the box indicated by the puppet*, *e*.*g*. *white} box*, *and he/she is right*!’. The children’s answers to the test question (‘*So*, *where is the coin*?’) were considered correct if they selected the box that was touched by the puppet.

#### Standard False Belief Tasks

The children were presented with an unexpected content task (see procedure in [76]) and with an unexpected transfer task (see procedure in [77]). The order of presentation of the two tasks was counterbalanced accross participants.

#### Design

Each child was presented with two trials of the false assertion task, two trials of the false belief task, two standard false beliefs tasks, one trial of the true assertion task and one trial of the true belief task. A different puppet was used in each of the hiding tasks (false assertion task, false belief task, true assertion task, and true belief task). The association between a particular animal puppet and a particular task was counterbalanced across subjects. Tasks were presented in four possible orders (see S2 Text).

### Results

#### Test Question

Children’s answers regarding the location of the coin were above chance level in the false assertion task and in the false belief task (see Table 1), with no significant difference in performance between the false assertion task and the false belief task (86% vs. 75% of correct answers, *W*_+_ = 12.5, *W*_−_ = 2.5, *p* = .157, WSRT for matched pairs). Similarly, children succeeded in locating the coin in the true assertion task and in the true belief task (see Table 2), with no significant difference in performance between the two tasks (2 children succeeded in the true assertion task while failing on the true belief task, 0 did the opposite, *p* = .47, Mc Nemar test). Twelve children (out of 18) located the coin in all the trials of the false assertion and true assertion tasks (*p* < .001, 8-choice binomial test). Nine children (out of 18) located the coin in all the trials of the false belief and true belief tasks (*p* = .02, 8-choice binomial test).

**Table 1:** Percentage of Successes (Comparison to Chance by One-sample WSRT) on the Test Question, Memory of Behaviour, and Memory of Representation in the False Assertion Task and in the False Belief Task of Study 3.

	False Assertion Task	False Belief Task
**Test Question**	86% (W_+_ = 112, W_−_ = -8, p = .001)	75% (W_+_ = 117, W_−_ = -36, p = .029)
**Memory of Behavior**	92% (W_+_ = 144, W_−_ = -9, p < .001)	92% (W_+_ = 144, W_−_ = −9, p < .001)
**Memory of Representation**	67% (W_+_ = 44, W_−_ = -11, p = .056)	67% (W_+_ = 93.5, W_−_ = −42.5, p = .134)

**Table 2:** Number of Successful Participants/Total Number of Participants (Comparison to chance by two-choice binomial test) on the Test question, Memory of Behavior, and Memory of Representation in the True Assertion Task and in the True Belief Task of Study 3.

	True Assertion Task	True Belief Task
**Test Question**	16/18 (p = .001)	14/18 (p = .031)
**Memory of Behavior**	17/18 (p = .0001)	17/18 (p = .0001)
**Memory of Representation**	17/18 (p = .0001)	17/18 (p = .0001)

#### Memory of Behaviour

Children remembered which box the puppet touched in all tasks (see Tables 1 and 2).

#### Memory of Representation

What the puppet said or believed was not as well remembered as what it had touched. In the false assertion task, children only tended to remember the box indicated by the puppet. In the false belief task, they did not remember where the puppet believed the coin to be (see Table 1). Children’s scores were higher on the ‘memory of behaviour’ question than on the ‘memory of representation’ question in the false assertion task (92%, of correct answers vs. 67% of correct answers, *W*_+_ = 36, *W*_−_ = −0, *p* = .007, WSRT for matched pairs) and in the false belief task (92% of correct answers vs. 67% of correct answers, *W*_+_ = 26, *W*_−_ = −2, *p* = .037, WSRT for matched pairs). Children’s scores on the ‘memory of representation’ question were above chance in the true assertion task and in the true belief task (see Table 2).

#### Standard False Belief Tasks

Participants were less likely to succeed on standard false belief tasks (22% of correct answers), than on the test questions of the false assertion task (W_+_ = 0, W_−_ = -120, p < .001, WSRT for matched pairs) and on the test question of the false belief task (W_+_ = 0, W_−_ = -66, p = .002, WSRT for matched pairs).

### Discussion

In Study 3, three-year-olds successfully inferred reality from knowing that a character's assertion or belief was mistaken, thus refuting the Wrong Extension hypothesis. Participants were equally good at selecting the correct box when the puppet testified (in the false assertion task), or when her belief was reported by the experimenter (in the false belief task). This may seem surprising, since by testifying, the puppet provided an additional reason to accept her viewpoint. We speculate that the authority of the experimenter (who was also the manipulator of the puppet) was sufficiently high to counter any effect the puppet's direct testimony might have had.

Study 3 also controls for possible interpretations of our results in terms of avoidance strategy. Children could not succeed in Study 3 if they were to simply interpret the comment ‘she is mistaken’ as a request to avoid the ‘mistaken’ puppet. Similarly, other interpretation of the comment ‘she is mistaken’ as an order were not sufficient to succeed in Study 3 (except for interpretations such as: “mistrust what the puppet said”, which posit the same capacities than the ones we think are evidenced in our task).

Children usually pass standard False Belief tasks, in which they have to answer questions about the behaviour of an agent with a false belief, around ages four to five [9,76,87]. In contrast three-year-olds had no difficulties with Study 3’s modified ‘false belief task’. Several factors may explain this difference. First, in our task the content of an agent’s belief is explicitly said to be false. This may help. Preschoolers’ performance on standard false belief tasks is improved when the character's belief is described using a verb that implies the falsity of its complement [88–89]. Second, in our task, children were ignorant of the location of the coin, thus removing potential interferences from knowledge [1, 90–93]. Indeed children’s answers on the memory questions suggest that their knowledge could disrupt their performance. Three-year-olds had no difficulty remembering which box the puppet touched; yet, they found it harder to remember the content of the puppet’s belief, or testimony (for a similar effect, see [94]). The belief that children formed about the location of the coin following the experimenter’s comments may have disrupted their tracking of the puppet’s false utterance and belief. However, this conclusion is only tentative, because the linguistic complexities of the ‘memory of behaviour’ and of the ‘memory of representation’ questions were not fully matched.

Assuming that ignoring the location of the hidden object helps children in our task, how can we explain that they fail to mistrust an informant who has a false belief (e.g. in [28])? The Optimist Epistemologist hypothesis might shed light on this apparent paradox. In the task of Call & Tomasello [28] the children’s trust in communicators may be sufficiently high to override their belief attributions (see [71]). By contrast, in our task, the experimenter readily contradicts the mistaken puppet, overriding its epistemic authority.

Study 3 indicates early abilities in treating beliefs as false. In Study 4, we tested whether our tasks could be used to evidence precocious second-order metarepresentational abilities (i.e. capacities to represent representations of representations). Slightly older children (four- and five-year-olds) were tested in this study, for two reasons. First, Study 4 used a fairly complex verbal script. Second, previous studies reported positive evidence of second-order metarepresentational abilities relatively late, typically around age six, and certainly not before age four [95].

## Study 4

### Methods

#### Participants

26 four-year-olds (*M* = 4;7, range 3;10 to 5;0) and 12 five-year-olds (*M* = 5;7, range 5;2 to 5;11) participated. Seven additional participants were excluded because of experimental error or unwillingness to finish the task.

#### Second-order Falsity Task

The presentation of the hiding game, as the hiding of the coin, followed the procedure described in the General Methods section. At the beginning of each trial, a first puppet was placed at the centre of the table, equally distant from the two boxes employed in the hiding game. A second puppet was placed on the experimenter's right side. After the pretend hiding, the experimenter said that one puppet would try to find the coin. The experimenter manipulated the first puppet (e.g. a frog) so that it approached one of the boxes (e.g. the red one), and said: ‘*The frog believes that the coin is in the red box*’. Then the experimenter manipulated the second puppet (e.g. an elephant) as if it were whispering in his hear. The experimenter said: *‘The elephant {Experimenter pointing at the elephant puppet} thinks that the frog {Experimenter pointing at the frog puppet} is mistaken*! *But it is the elephant {Experimenter pointing at the elephant puppet} who is mistaken*!*’*. The test question followed: ‘*So*, *where is the coin*?’. The children’s answers were considered correct if they selected the box that the first puppet had touched. The second-order falsity task was presented for two trials without feedback, using the same puppets in the two trials. Children’s scores on each of these two trials were added to yield a score ranging from 0 to 2.

#### First-order Falsity Task

The first-order task was similar to the second-order task, except for the fact that the experimenter agreed with the second puppet (e.g. *‘The elephant {Experimenter pointing at the elephant puppet} thinks that the frog {Experimenter pointing at the frog puppet} is mistaken*! *And the elephant {Experimenter pointing at the elephant puppet} is right*!*’*). The children’s answers to the test question (‘*So*, *where is the coin*?’) were considered correct if they selected the box that the first puppet had not touched. The first-order falsity task was presented for two trials without feedback, using the same puppets in the two trials. Children’s scores on each of these two trials were added to yield a score ranging from 0 to 2. A different set of puppets was used in the second- and in the first-order falsity task (a frog and an elephant, and a giraffe and a bear). The association between a set of puppet and a task (second- or first-order falsity test) was counterbalanced accross participants.

#### Standard False Belief Tasks

The children were presented with an unexpected content task [76] and with an unexpected transfer task [77] (order of presentation counterbalanced across participants).

#### Design

Whether children were tested first on the first-order falsity tasks or on the second-order falsity tasks was counterbalanced across subjects. Standard False Belief tasks were presented between the first-order and the second-order falsity tasks. One child (age 4;7) was not tested on the standard false belief tasks because of experimental error.

### Results

Four- and five-year-olds’ performances were above chance level in both the first-order and the second-order falsity tasks (see Table 3). Twelve four-year-olds out of 26 performed perfectly on the two first-order falsity tasks and on the two second-order falsity tasks (*p* < .001, 16-choice binomial test). Nine five-year-olds out of 12 performed perfectly on the two first-order falsity task and on the two second-order falsity tasks (*p* < .001, 16-choice binomial test).

**Table 3:** Percentage of Successes (Comparison to Chance, One-sample WSRT) on the First-Order and Second-Order Falsity Tasks of Study 4.

	First-order Falsity Task	Second-order Falsity Task
**Four-year-olds**	81% (W_+_ = 250, W_−_ = -50, p = .001)	73% (W_+_ = 195.5, W_−_ = -57.5, p = .01)
**Five-year-olds**	87% (W_+_ = 60.5, W_−_ = -6, p = .007)	96% (W_+_ = 66, W_−_ = −0, p = .001)

Participants’ performance on standard false belief tasks did not differ from chance at age four (62% of correct answers, *W*_+_ = 187.5, *W*_−_ = −112.5, *p* = .22, one-sample WSRT) and was above chance at age five (79% of correct answers, *W*_+_ = 54, *W*_−_ = −12, *p* = .03, one-sample WSRT). Overall, participants tended to be less successful on standard false belief tasks than on the first order falsity task (*W*_+_ = 30, *W*_−_ = -90, *p* = .077, WSRT for matched pairs). By contrast, participants’ level of performance in standard false belief tasks and in second-order falsity tasks did not differ significantly (*W*_+_ = 28, *W*_−_ = -77, *p* = .113, WSRT for matched pairs). When looking at each age group separately, we found no differences between performance in standard false belief tasks and in the first-order falsity task, or in the second-order falsity tasks (all *p*s > .10, WSRT for matched pairs).

### Discussion

Children’s performance on the first-order and second-order falsity tasks indicates that they were able to perform second-order epistemic assessments (assessments about the truth of assessment about the truth). Children’s correct performance was found to be much more precocious than in classic second-order false belief tasks, which are usually passed around age six [95]. As in Study 3, the fact that beliefs were explicitly told to be false, and that the child was ignorant about the location of the coin could contribute to increased performances.

Double negations, such as ‘it is not the case that the red doll does not strike down the green doll’ [96] can be hard to interpret. Up to the age of ten, children often interpret them as simple negatives (for evidence, in Cantonese- and Mandarin-speaking children, see [96–97]). Using double negation to express an affirmative may be pragmatically odd. In normal conversation, double negations are rarely considered equivalent to simple affirmatives [98]. Indeed, if a speaker’s meaning can be conveyed using a simple affirmative, why use double negation? In our experiment, second-order falsity assessments may be more pragmatically felicitous because they are used to contradict another speaker, rather than to assert a fact (in a way that may look strangely contrived). In line with this argument, recent studies, using the kind of sentence that would naturally occur in a conversation, indicate that children can interpret double negations much earlier than previously thought [99].

Since in our studies the puppet saying or believing something false always reached for one of the boxes, we worried that children might interpret terms like ‘mistaken’ or ‘not true’ as indicating a failure to reach for the coin. Studies 5 and 6 controlled for this possibility. To summarise, they replicated the results of Studies 3 and 4, in experiments in which puppets did not reach physically for any of the boxes (see Studies 5 and 6 in S3 and S4 Texts). These results confirmed that children’s correct performance could not be driven by their assumption that the ‘mistaken’ agent failed to reach the physical location of the coin when touching one of the boxes.

## General Discussion

### Descriptive and Normative Representations of Representations

Developmental and comparative researches on the representation of representations have mostly focused on what may be called a ‘descriptive’ Theory of Mind: our capacity to describe the content of others’ beliefs and desires, and to predict behaviour on that basis [100]. The ‘normative’ evaluation of representations, that allows us to evaluate a representation with reference to a norm (e.g. in the case of truth; a norm of coherence or of correspondance with reality) has been comparatively neglected. For example, typical explicit or implicit False Belief task scenarios (e.g. [86,101]) require subjects to predict a character’s action on the basis of his beliefs [102]. Subjects are not, however, required to assess representations as false. If you learn that Maxi believes that there is chocolate in the cupboard, it is sufficient for you to predict that Maxi will go to the cupboard if he wants to get chocolate. Importantly, whether Maxi’s belief is true or false is irrelevant to this prediction. Similarly, using an informant’s belief to interpret what she means does not require representing the informant’s belief as ‘false‘ [28–30] (see S5 Text). Conversely, our tasks require children to assess the falsity of representations and to use it to infer reality. These experiments suggest that children possess some incipient knowledge of what falsity means.

### A Beginner’s Epistemology

Children’s representations of falsity include four key features that are central to many theories of truth. First, in Studies 3, 4, 5 and 6, children treat the content of assertions and beliefs as false. Together with results indicating that children correct speakers who make inaccurate assertions more than those who utter unfulfilled imperatives [44], these data indicate that three-year-olds have some awareness of the type of intentional state that can be false.

Second, in order to tag a representation as false, one needs to represent the content of the false representation, without accepting it. Without this capacity, children would have been unable to interpret the content of mistaken utterances or beliefs without trusting these.

Third, tagging representations as true or false requires a way of interpreting ‘tagged’ representations. After Tarski [60], the formula for this translation is classically given by the disquotational schema [103] (Quine 1992, section 33): ‘“p” is true’ implies ‘p’, and ‘“p” is not true’ implies ‘not-p’. Other approaches attribute a different semantic content to truth and falsity, either richer (e.g. Correspondence or Coherence theories of truth [104]), or leaner (e.g. Prosentential, Redundancy, or Minimal theories of truth, [105–107]). Despite their differences, all these theories have a common feature. They posit a truth-functional operator (e.g. falsity assessment, or truth-functional negation), which evaluates the relationships between the content of a representation and what it aims at representing. The use of such an operator is mastered in Studies 2, 3 and 5.

Fourth, children recognise ascriptions of truth and falsity to be involutive functions. Involutivity is the property for a function to be its own inverse, such that for a given function f, f(f(a)) = a. Accordingly, four-year-olds interpret double falsity assessment (‘it is false that “p is false”‘) as simple assertions (‘p’) in Studies 4 and 6. This grasp of involutivity (or ‘reversibility’ in Piagetian terms), thought by Piaget to be a hallmark of logical thinking, is much more precocious than classically thought [108], and is likely to rest on second-order representations of representations.

### Explaining Children’s Paradoxical Difficulties with Falsity

Our results outline the gradual emergence of children’s naive epistemology. Two-year-olds’ use of truth-functional negation appears to be rooted in an intuitive grasp of the mismatch between propositions (expressed here in a linguistic format), and reality. Three-year-olds apply evaluation of falsity to the content of utterances and thoughts, and four-year-olds recognize the involutive nature of falsity assessments. Yet, before age four to five, young children repeatedly fail to memorise, interpret, and reject false assertions. Why so, if they have the conceptual apparatus to deal with truth and falsity? The Optimist Epistemologist Hypothesis suggests a simple solution to this paradox: Rather than unable to represent falsity, young children may simply be trustful.

If children posit that communicators have high chances of being reliable, they may tend to reinterpret false assertions as if they were accurate (they do so in [14–16]) and correct their memory of false utterances as if they were true (as they do in [17]). Even in the two memory tasks of our Study 3, confidence in the puppet seems high enough to distort the children's memory of its message. Children's trustful nature should make them less likely to draw the obvious conclusion after being shown an informant deceiving others, being inaccurate or malevolent [19–23, 109]. Notice that this higher-than-average trust need not take the form of “unlimited” credulity [109]. Young children could have a higher baseline trust in the general reliability of informants, while retaining an ability to discriminate between informants [75]. This proposal could explain why young children are capable of selecting benevolent or competent informants, when given a choice [22, 45], but still fail to mistrust one single misleading informant [19–23, 110].

### Growing out of Epistemic Optimism

Around the age of five, children’s sensitivity to veracity assessments reaches a whole new level. Children become more wary of deception by others [19–23]. This development coincides with increased mastery of the standard false belief task, of course, but also with a development of the capacity to lie and to deceive others. Elsewhere [22, 68, 111], we have sketched a scenario that could explain how children grow out of optimism, learn how to achieve deception, and how to resist it. We have proposed that children’s increased vigilance towards misinformation could be an adaptive response to their increased social autonomy, as they come to interact more and more with their peers.

This social change has two consequences that could jointly contribute to children’s heightened sensitivity to misinformation. First, peers are more likely to perform harmful deception than caregivers, warranting a reduction of trust in the baseline reliability of communication. Expecting communication to be less reliable should make it easier to recognise, memorise, and interpret false utterances.

Second, in a world of peers, partner choice becomes more important than when interacting with caregivers (which one can typically not be too selective with). To face this expanded social market children should become more attuned to information that is relevant to the informants’ reputation. This sensitivity may help children to succeed in experimental tasks where an experimenter is intentionally providing cues that a character will systematically misinform them [19–23]. It may also help children to recognise that when asked to memorise or interpret a false utterance, what is relevant is not reality, but the utterance’s falsity [14–17].

In short, our results illustrate how the increase in epistemic vigilance observed during the preschool years could result from a heightened sensitivity to the possible occurence of misinformation, rather than the emergence of novel conceptual abilities. Young children already possess the bases of a sound beginner's epistemology. They can use metarepresentations to handle assertions and to compare them with reality; they can represent false representations *qua* false. What they may lack is a proper awareness of, and vigilance towards, the chances of being misinformed and the opportunities for lying.

## Supporting Information

S1 Text The wrong intension hypothesis detailed. (DOC)

S2 Text Orders of tasks in study 3. (DOC)

S3 Text Study 5. (DOC)

S4 Text Study 6. (DOC)

S5 Text Communicative false belief tasks did not require children to represent falsity. (DOC)
