# Supplementary material for: Epistemology for Beginners: Two- to Five-Year-Old Children's Representation of Falsity
Source: PLoS One. 2015 Oct 20;10(10):e0140658. doi: 10.1371/journal.pone.0140658 (PMC4618725; doi:10.1371/journal.pone.0140658)
Supplement: S1 Text — (DOC) [file pone.0140658.s001.doc]

S1 Text. The Wrong Intension Hypothesis Detailed

According to the Wrong Intension Hypothesis, children before the age of four fail to grasp what it means for a proposition to be false. This is not to say that children are unable to evaluate a message's content or to select the best of two informants. Their capacity to discriminate between sources of information, while real, would not rest on representations of falsity or truth. Truth-conditional content is only one of the possible grounds for evaluating assertions and informants, and for filtering available information. Other criteria include:

— Informativeness. A cognitive system may simply devote fewer resources to stimuli and messages coming from sources of information it has learnt to disregard as uninformative. Vervet and rhesus monkeys respond to calls, but they are less responsive when the caller repeatedly produced misleading calls in the past [1–2]. Should we assume that monkeys’ selective learning is based on treating these alarm calls as false? Not necessarily. A cognitive system can block learning from certain sources without attaching a ‘true’ or a ‘false’ tag to the representations that it handles. The trick is simply to learn to use certain sources of information but not others . Obviously, this computational strategy works up to a point only; but it has one clear benefit. It does away with the need for metarepresentational abilities [3]. Imagine a case in which a speaker is mislabelling a familiar object (e.g. saying ‘It’s a cup’ while pointing at a shoe). The speaker’s meaning conveys the presence of an object (a cup), while this object is absent. Noticing such a discrepancy would be sufficient for children to treat the speaker as ‘uninformative’, i.e., as producing cues that are not correlated with what they are supposed to inform about (a property also sometimes called ‘unreliability’, see [4]). Young children could treat inaccurate assertions as uninformative rather than false. This would give them reason enough to correct inaccurate informants, and reduce their reliance on those.

— Processing costs. The phrase ‘The time of the day is equal to three fifth by twenty-five third minus square-root of sixteen hours’ and the phrase ‘It is one o'clock’ have the same truth value, yet the second should obviously be preferred on grounds of cognitive efficiency [5-6]. Arguably, some mistakes are harder to interpret than accurate instances of communication [7]. One relevant case is mislabelling, calling one thing by an inaccurate name. Mislabelling makes it harder to disambiguate the referent of a speaker’s communicative action, and to establish her meaning. Young children may evaluate such inaccurate assertions in a negative manner, not because they recognise their falsity, but because they find them harder to interpret.

— Linguistic Adequacy. A statement can be evaluated on formal grounds that are independent from the truth of what the speaker asserts, as in the following example (adapted from [8]):

John: We saw hippopotamuses.

Mary: No. We saw hippopotami.

In this case, Mary does not challenge the truth of John’s assertion. She questions, however, the appropriateness of John’s use of the plural morphology. This type of ‘metalinguistic’ assessment has been hypothesised to account for many instances of young children’s use of negation ([9-10]).

In sum, sensitivity to informativeness, processing costs, or linguistic appropriateness could be sufficient to identify inaccurate assertions, to correct them, and to lower one's trust in inaccurate communicators.

References

1. Cheney DL, Seyfarth RM. How monkeys see the world. Chicago, IL:

Chicago University Press; 1990.

2. Gouzoules H, Gouzoules S, Miller K. Skeptical responding in rhesus monkeys (Macaca mulatta). Int J Primatol. 1996;17:549–68.

3. Cosmides L, Tooby J. Consider the source: The evolution of adaptations for decoupling and metarepresentation. In: Sperber D, editor. Metarepresentations: A multidisciplinary perspective. NY: Oxford University Press; 2000. p.53–115.

4. Searcy, WA, Nowicki S. The evolution of animal communication: reliability and deception in signaling systems. Princeton University Press; 2005.

5. Guilford T, Dawkins MS. Receiver psychology and the evolution of animal signals. Anim Behav. 1991;42:1-14.

6. Sperber D, Wilson D. La pertinence: Communication et cognition (French translation of Relevance: Communication and cognition). Paris: Les Editions de Minuit; 1989.

7. Wilson D. Metarepresentation in linguistic communication. In: Sperber D, editor. Metarepresentations: A multidisciplinary perspective. Oxford: Oxford University Press; 2000. p. 411-443.

1. Carston R. Metalinguistic negation and echoic use. J Pragmat. 1996;25: 309-330.

9. Drozd KF. Child English pre-sentential negation as metalinguistic exclamatory sentence negation. J Child Lang. 1995;22:583-610.

10. Horn LR. A natural history of negation. Chicago: University of Chicago Press; 1989.
