# Supplementary material for: Epistemology for Beginners: Two- to Five-Year-Old Children's Representation of Falsity
Source: PLoS One. 2015 Oct 20;10(10):e0140658. doi: 10.1371/journal.pone.0140658 (PMC4618725; doi:10.1371/journal.pone.0140658)
Supplement: S2 Text — (DOC) [file pone.0140658.s002.doc]

S2 Text. Orders of Tasks in Study 3

In Study 3, tasks were presented in four possible orders counterbalanced across subjects : 1. false assertion tasks, false belief tasks, standard false belief tasks, true assertion tasks, true belief tasks (five participants); 2. true assertion tasks, true belief tasks, standard false belief tasks, false assertion tasks, false belief tasks (five participants); 3. false belief tasks, false assertion tasks, standard false belief tasks, true belief tasks, true assertion tasks (four participants); 4. true belief tasks, true assertion tasks, standard false belief tasks, false belief tasks, false assertion tasks (four participants).
