# Supplementary material for: Epistemology for Beginners: Two- to Five-Year-Old Children's Representation of Falsity
Source: PLoS One. 2015 Oct 20;10(10):e0140658. doi: 10.1371/journal.pone.0140658 (PMC4618725; doi:10.1371/journal.pone.0140658)
Supplement: S3 Text — (DOC) [file pone.0140658.s003.doc]

S3 Text. Study 5

Methods

Participants

Twenty three-year-olds (M = 3;7, range 3;1 to 4;0) participated.

Procedure

Participants were presented with the same tasks as in Study 3, counterbalanced in the same ways (five participants were tested in each of the four possible counterbalancing orders described in text S2). However, the puppet did not touch any of the boxes in any of the hiding tasks (false assertion task, false belief task, true assertion task, and true belief task). Instead, the puppet was left equidistant from both boxes during the whole experiment. Since the puppet did not touch any box, no ‘memory of behaviour’ question was asked.

Main Results

Three-year-olds’ results showed the same pattern of significant results as in Study 3, with one exception. They performed above chance levels in the ‘memory of representation' question of the false assertion task (75% of correct answers, W_+_ = 110.5, W_−_ = −25.5, p = .01, one-sample WSRT), and of the false belief task (77% of correct answers, W_+_ = 104, W_−_ = −16, p = .005, one-sample WSRT). The differences in performance between Study 3 and Study 5, on these two questions, were not significant. Detailed results are presented below.

Test Question

Children succeeded in locating the coin in the false assertion task (77% of correct answers, W_+_ = 112, W_−_ = −8, p = .008, one-sample (WSRT)) and in the false belief task (75% of correct answers, W_+_ = 117, W_−_ = −36, p = .01, one-sample WSRT). Similarly, children succeeded in locating the coin in the true assertion task (18 children out of 20, p = .0004, two-choice binomial test), and in the true belief task (17 children out of 20, p = .003, two-choice binomial test). Thirteen children (out of 20) located the coin in all the trials of the false assertion and true assertion tasks (p < .001, 8-choice binomial test). Twelve children (out of 20) located the coin in all the trials of the false belief and true belief tasks (p < .001, 8-choice binomial test).

Memory of Representation

Children performed above chance levels on the ‘memory of representation’ question of the false assertion task (75% of correct answers, W_+_ = 110.5, W_−_ = −25.5, p = .01, one-sample WSRT), and of the false belief task (77% of correct answers, W_+_ = 104, W_−_ = −16, p = .005, one-sample WSRT). The differences in performance between Study 3 and Study 5 on these two questions were not significant. Children’s scores on the ‘memory of representation’ question were above chance in the true assertion task (18 correct children out of 20, p = .0004, two-choice binomial tests) and in the true belief task (20 correct children out of 20, p < .0001, two-choice binomial tests).

Standard False Belief Tasks

Children’s scores on standard false beliefs tasks (12.5 % of correct answers) were lower than their scores on the test question of the false assertion task (W_+_ = 136, W_−_ = 0, p = .0003, WSRT for matched pairs) and on the test question of the false belief task (W_+_ = 136, W_−_ = 0, p = .0003, WSRT for matched pairs).
