# Supplementary material for: Epistemology for Beginners: Two- to Five-Year-Old Children's Representation of Falsity
Source: PLoS One. 2015 Oct 20;10(10):e0140658. doi: 10.1371/journal.pone.0140658 (PMC4618725; doi:10.1371/journal.pone.0140658)
Supplement: S4 Text — (DOC) [file pone.0140658.s004.doc]

S4 Text. Study 6

Methods

Participants

Sixteen four-year-olds (M = 4;7, range 4;1 to 5;2) participated. One additional four-year-old refused to participate in the full experimental session and was excluded from analysis.

Procedure

Participants were presented with the same tasks as in Study 4, but puppets never approached the boxes during the experiment. The experimenter reported the belief of a first puppet by saying e.g. : ‘The frog believes that the coin is in the white box’. A second puppet was then introduced, and the experiment proceeded as it did in Study 4. The second puppet whispered in the experimenter’s ear while remaining on his right side. The box that children had to select to answer the test question correctly was on the left in half of the trials, on the right in the other half. Therefore, it was closer to the second puppet in half the trials, and further from it in half of the trials. Other counterbalancings were similar as in Study 4.

Results

Results showed the same pattern of significant results as in Study 4. Children’s performances were above chance in the first-order falsity task (72% of correct answers, W+ = 54, W− = −12, p = .039, one-sample WSRT) and in the second-order falsity task (78% of correct answers, W+ = 77, W− = −14, p = .015, one-sample WSRT). Five four-year-olds out of 16 performed perfectly on the two first-order falsity task and on the two second-order falsity tasks (p =.003, 16-choice binomial test). Participants’ level of performance in standard false belief tasks (53% of correct answers) was not significantly different from their level of performance in the first-order falsity tasks (W_+_ = 9, W_−_ = -27, p = .196, WSRT for matched pairs) or in the second-order falsity task (W_+_ = 18.5, W_−_ = -47.5, p = .182, WSRT for matched pairs).
