# Supplementary material for: Epistemology for Beginners: Two- to Five-Year-Old Children's Representation of Falsity
Source: PLoS One. 2015 Oct 20;10(10):e0140658. doi: 10.1371/journal.pone.0140658 (PMC4618725; doi:10.1371/journal.pone.0140658)
Supplement: S5 Text — (DOC) [file pone.0140658.s005.doc]

S5 Text. Communicative False Belief Tasks Did Not Require Children to Represent Falsity.

Some false belief tasks have used a setting roughly comparable to our false assertion and false belief tasks [1-3]. In these experiments, children had to infer reality from what an informant with a false belief communicated to them. To pass those tasks, children needed to interpret what a communicator meant by representing the content of her (false) belief. However, children did not need to represent the falsity of the belief itself. For example, in a study by Call and Tomasello [1], children were presented with a hiding game in which they had to find a reward hidden in one of two containers. An experimenter (the communicator) tried to help participants by placing a marker on the container that she believed to hold the reward. In the false belief trials, the communicator watched the hiding process and then left the area, at which time a second experimenter switched the locations of the containers. When the communicator returned, she marked the container at the location where she had seen the reward hidden, i.e. the incorrect one. In the words of Call and Tomasello, “Successful performance required participants to reason as follows: the communicator placed the marker where she saw the reward hidden; the container that was at that location is now at the other location; so the reward is at the other location.” To find the reward in this task, children thus needed to establish that the informant believed that the container on which she placed the marker was the one where she saw the reward being placed. This computation required to represent the agents’ belief, but not to evaluate this belief as ‘false’.

References

1. Call J, Tomasello M. A nonverbal false belief task: The performance of children and great apes. Child Dev. 1999;70:381–395.

2. Happé F, Loth E. ‘Theory of mind’and tracking speakers’ intentions. Mind Lang. 2002;17:24–36.

3. Southgate V, Chevallier C, Csibra G. Seventeen‐month‐olds appeal to false beliefs to interpret others’ referential communication. Dev Sci. 2010;13:907–912.
